# Interaction of Tamoxifen Analogs With the Pocket Site of Some Hormone Receptors. A Molecular Docking and Density Functional Theory Study

**DOI:** 10.3389/fchem.2018.00293

**Published:** 2018-07-13

**Authors:** Linda-Lucila Landeros-Martínez, Daniel Glossman-Mitnik, Norma Flores-Holguín

**Affiliations:** Laboratorio Virtual NANOCOSMOS, Departamento de Medio Ambiente y Energía, Centro de Investigación en Materiales Avanzados, Chihuahua, Mexico

**Keywords:** tamoxifen analogs, density functional theory, chemical reactivity, M06 functional, oxidative damage

## Abstract

In this paper, the antiestrogenic properties of Tamoxifen analogs have been investigated and a theoretical report of its analogs interaction with the pocket site of some hormone receptors are presented. Analogs were generated by modification of the hydrophilic functional group of Tamoxifen by hydroxyl, amide, carboxyl, and sulfhydryl functional groups, in an attempt to improve their activity and selectivity. The analogs exhibit a negative binding energy in the estrogen and progesterone receptors, which indicates a spontaneous interaction between the analogs and the pocket site in the hormone receptors. The values of the molecular polar surface area indicate that the analogs have good permeability and are strong electrophiles. The couplings showed electrostatic interactions such as hydrogen bond and π-π interactions. According with the Lipinsky Rule of Five, the four analogs presented a good biodistribution, permeability, and pharmacological action on the hormone receptors. The analysis of the charge transfer suggests a limited enhanced oxidative damage in the estrogen receptor that not takes place with the progesterone receptor.

## 1. Introduction

Tamoxifen (TAM) is a drug widely prescribed as chemopreventive for women to prevent and to treat all stages of breast cancer (Jordan, 2007; Esteve-Romero et al., 2010). TAM is a Selective Estrogen Receptor Modulator (SERM) (Boyd and Coner, 1996; Jordan, 2003), which acts as a blockage for the effects of estrogen in the breast tissue by attaching to the estrogen receptors in breast cells.The targets for this drug are some hormone receptors [estrogen receptors (ER) and progesterone receptors (PR)]. This drug is a prodrug and can be metabolically activated to 4-hydroxytamoxifen (4OHTAM) (Jordan et al., 1977; Borgna and Rochefort, 1981) or alternatively can be metabolically routed via N-desmethyltamoxifen (NDTAM) to 4-hydroxy-N-desmethyltamoxifen also known as endoxifene (END) (Irarrazával, 2011; Sanyakamdhorn et al., 2016). The hydroxyl metabolites of tamoxifen have a high binding affinity for the ER (Jordan et al., 1977).

The recent exponential growth of computational resources has facilitated successful development of theoretical algorithms that can also be used to study the electronic effects (Brewerton, 2008). These algorithms can also be used to calculate other physical and chemical properties of ligands using semiempirical and Density Functional Theory (DFT) methods (Correa-Basurto et al., 2012). The theoretical results obtained with these methods have been successfully compared with experimental results (Ravna et al., 2007).

A huge amount of theoretical studies on TAM has already been carried out to describe its interaction with ER. Using calculations of molecular dynamics, semiempirical, and DFT in conformational analysis of TAM and Toremifene (TOR), it was predicted that TOR conformations were slightly different from those of TAM owing to the effect of the chlorine atom at chloroethyl group (Kuramochi, 1996). In a recent research, Landeros-Martinez et al. analyzed the molecular docking of TAM in ER and PR in which the active site of the hormone receptors were determined, as well as the charge transfer of the TAM to the residues of the active sites in the hormone receptors (Landeros-Martínez and Flores-Holguín, 2016). Other theoretical studies analyzed the metabolism of TAM using semiempirical (PM3) and DFT with B3LYP/6-31G^*^ methods (Hariharan and Pople, 1973; Francl et al., 1982). Also a study of the molecular conformations and the vibrational NMR spectra of TAM performed with B3LYP/6-311(d,p) has been reported (Badawi and Khan, 2016). Another theoretical IR and ultraviolet-visible (UV-Vis) spectra of TAM drug were compared with the experimental data where the methodology that have been found in a better correlation with experimental data is M06/6-31G(d) (Landeros-Martínez et al., 2017).

On the other hand, the molecular docking is an operation in which one molecule is brought into the vacancy of another while calculating the interaction energies of the numerous mutual orientations and conformations of the two interacting species (Bultinck et al., 2003). This technique allows predicting the preferred conformations of a molecule, being bonded to another (Lengauer and Rarey, 1996), and it is widely used in drug design (Kitchen et al., 2004). Mathew et al. have employed a molecular docking procedure to estimate the analogs of TAM and Reloxifen (REL) with high affinity, which could be considered a possible lead molecule for drug design (Mathew and Raj, 2009).

The aim of this work is to modify the hydrophilic functional groups of the TAM by the hydroxyl, amide, carboxyl, and sulfhydryl functional groups to achieve better activity improvement and selectivity. These analogs were studied to determine the binding activity into the hormone receptor by molecular docking and DFT analysis that allowed to decide which analog generates more oxidative damage at the active site. Also, the study of the molecular polar surface area (PSA) permitted to quantify if Tamoxifen analogs (TAM-analogs) have good permeability in cell.

## 2. Settings and computational methods

### 2.1. Optimization, frontier molecular orbitals, and electronic structure calculations

The optimized structures of the different TAM-analogs were calculated by means of the hybrid meta-GGA M06 density functional (Zhao and Truhlar, 2008a,b) developed by the Truhlar group from the University of Minnesota, combined with the 6-31G (d) basis set proposed by the Pople group (Hariharan and Pople, 1973; Francl et al., 1982) and the continuous polarizable solvent model (CPCM) (Tomasi and Persico, 1994) using water as a solvent. The latter was used to obtain the Highest Occupied Molecular Orbital (HOMO) and Lower Unoccupied Molecular Orbital (LUMO) of each of the analogs, respectively. These calculations were carried out using the Gaussian 09 suite of programs (Frisch et al., 2018). The energy calculations of the amino acids that make up the active site on the estrogen, progesterone and TAM-analogs as well as the chemical reactivity descriptors are calculated with the M06/6-31G(d) model chemistry and CPCM using water as a solvent. All calculations were performed using DFT (Hohenberg and Kohn, 1964; Kohn and Sham, 1965; Parr and Yang, 1989). The charge distributions for the amino acids and TAM-analogs were obtained through the Hirshfeld population analysis (Hirshfeld, 1977).

Density functional methodology provides an excellent framework to define a set of known chemical concepts such as ionization potential (I) (Foresman and Frisch, 1996; Lewars, 2003), electron affinity (A) (Foresman and Frisch, 1996; Lewars, 2003), chemical hardness (η) (Parr and Pearson, 1983; Parr and Yang, 1984), electronegativity (χ) (Parr and Pearson, 1983; Parr and Yang, 1984), and electrophilicity (ω) (Parr et al., 1999). These reactivity descriptors were obtained by means of energy difference calculations. The chemical hardness, electronegativity, and electrophilicity are defined as:

(1) $$\begin{array}{l} \eta =\frac{1}{2}\left(I-A\right)\approx \frac{1}{2}\left(\epsilon_{L}-\epsilon_{H}\right) \end{array}$$

(2) $$\begin{array}{l} \chi =-\mu =\frac{1}{2}\left(I+A\right)\approx \frac{1}{2}\left(\epsilon_{L}+\epsilon_{H}\right) \end{array}$$

(3) $$\begin{array}{l} \omega =\frac{\mu^{2}}{2\eta }=\frac{{\left(I+A\right)}^{2}}{4\left(I-A\right)}\approx \frac{{\left(\epsilon_{L}+\epsilon_{H}\right)}^{2}}{4\left(\epsilon_{L}-\epsilon_{H}\right)} \end{array}$$

where μ is the chemical potential (Parr and Pearson, 1983; Parr and Yang, 1984) and ϵ_*H*_ and ϵ_*L*_ are the energies of the HOMO and LUMO, respectively.

The overall interaction between the TAM-analogs and the amino acids that make up the active site on ER and PR can be quantified through the charge transfer between the chemical species. This parameter determines the behavior of the different molecular systems as a donor or as an acceptor system. In this case, the electrons were transferred from the TAM-analogs to the amino acids of the active site of receptors or vice versa. The global interactions between two constituents has been calculated using the charge transfer parameter (ΔN) which is given by Padmanabhan et al. (2007):

(4) $$\begin{array}{l} \Delta N=\frac{\mu_{B}-\mu_{A}}{2\left(\eta_{A}+\eta_{B}\right)} \end{array}$$

The molecular polar surface area (PSA) was obtained through Molinspiration, a free software readily available on the Web (Molinspiration, 2018). To obtain PSA, the TAM-analogs were encoded with SMILES (Simplified Molecular Input Line System), which is a chemical notation system designed for modern chemical information processing (Weininger, 1988).

### 2.2. Molecular docking

The crystal structures of the estrogen and progesterone receptor were retrieved from the Protein Data Bank PDB: 1A52 and 1A28 respectively. The molecular docking was calculated with the specially tailored AutoDock 4.2 software with the Lamarckian Genetic Algorithm (LGA) (Morris et al., 2009) to explore how ER and PR bond with the TAM analogs. The water molecules in the receivers were eliminated and only the H-atoms polar were added. The docking area is selected by constructing a grid box of size 52 × 36 × 34 points, centered at x, y, and z coordinates of 89.304, 14.745, and 70.512, respectively for ER, and for PR, the grid box size 20 × 18 × 26 points was centered at x, y, and z coordinates of 36.999, 31.767, and 42.694, respectively, using in both receptors a grid spacing of 0.375 Å in AutoGrid (Morris et al., 2009). The docking parameters used for the LGA based conformational searches are docking trials: 150, population size: 150, maximum number of energy evaluations: 25000000, maximum number of top individuals to survive to next generation: 1, rate of gene mutation: 0.02, rate of crossover: 0.8: Mean of Cauchy distribution for gene mutation: 0.0, variance of Cauchy distribution for gene mutation: 1.0, and number of generations for picking the worst individual: 10.

## 3. Results and discussion

### 3.1. Analysis of analogs of tamoxifen

#### 3.1.1. Geometry optimization, frontier molecular orbitals, and electrostatic potential surface

**Figure 1:**
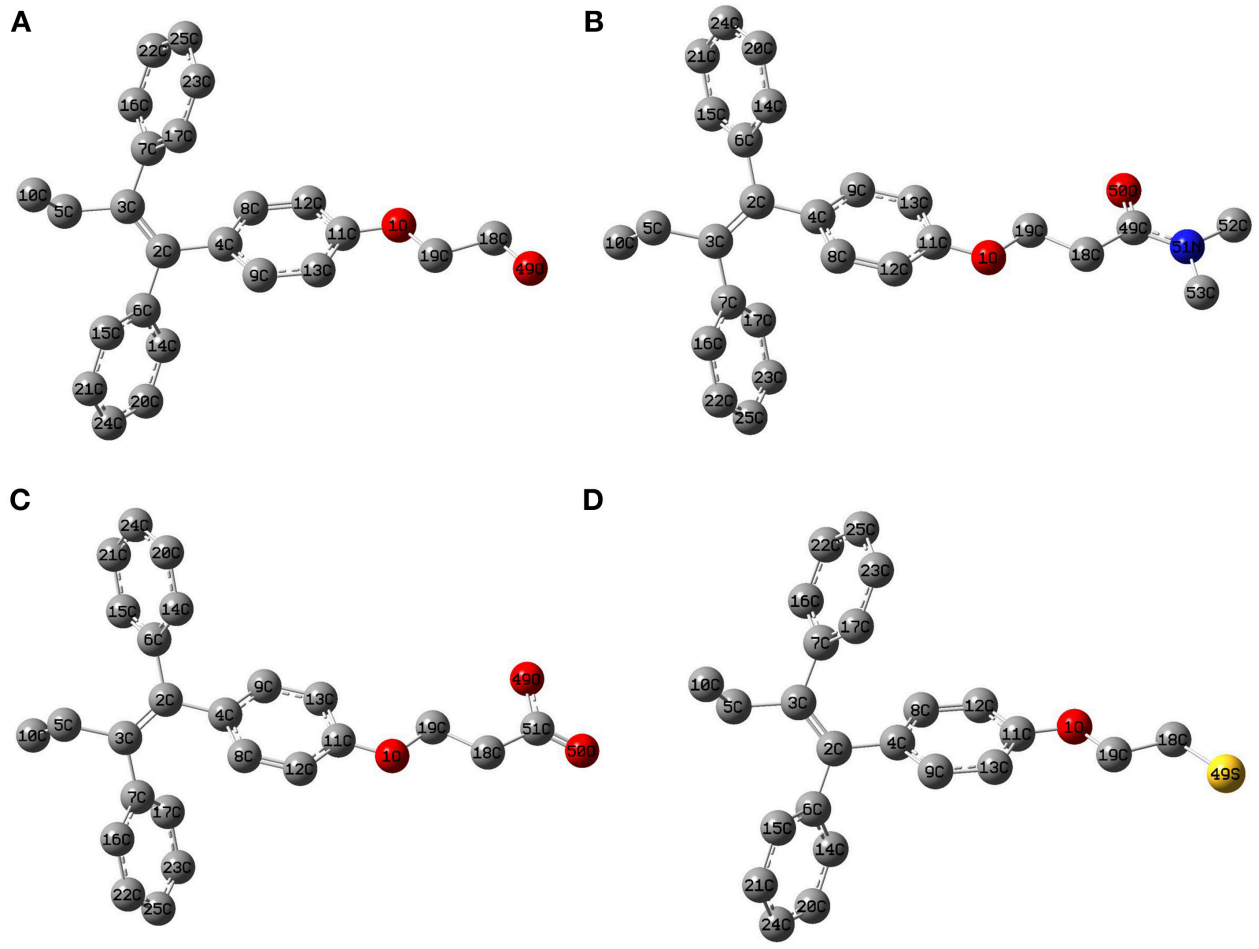
Optimized molecular structure of the Tamoxifen analogs at the M06/6-31G(d) level of theory: **(A)** TAM-Hydroxyl; **(B)** TAM-Amide; **(C)** TAM-Carboxyl; **(D)** TAM-Sulfhydryl.

The geometry optimization and frequency calculation of the TAM-analogs were performed to make sure that the molecules were at their lowest energy level. Figure 1 shows the optimized geometries of the studied molecules. The optimized TAM-analogs show a non-planar geometry due to the four dihedral angles in their structures as we can see in Table 1. A small difference in the dihedral angles has been observed in comparison with the TAM drug reported by Landeros-Martínez et al. (2017): there is an average difference of 0.68 degrees in DA, 1.021 degrees in DA2 and 0.01 degrees in DA3. Moreover, the dihedral angles DA4, DA5, DA6, and DA7 that were found on the opposite end of the TAM-analogs were 179.87 degrees, −179.77 , 179.06, and 178.99 degrees respectively. These dihedral angles have a greater differences compared to the TAM drug results (Landeros-Martínez et al., 2017). The values for the cartesian coordinates belonging to the optimized molecular structures of all the analogs are presented within the Supplementary Materials.

**Table 1:** Dihedral angles (°) of the Tamoxifen analogs determined at the M06/6-31G(d) level of theory.

**Bonding atoms**	**Dihedral angles (**°**)**			
	**TAM-Hydroxyl**	**TAM-Amide**	**TAM-Carboxyl**	**TAM-Sulfhydryl**
DA1 (4C-C2-6C-15C)	127.10	127.25	126.13	126.85
DA2 (4C-C2-3C-7C)	−9.21	−9.90	−9.44	−9.60
DA3 (4C-C2-3C-5C)	171.85	171.28	171.87	171.85
DA4 (1O-19C-18C-49O)	179.87	—	—	—
DA5 (1O-19C-18C-49C)	—	−179.97	—	—
DA6 (1O-19C-18C-51C)	—	—	179.06	—
DA7 (1O-19C-18C-49S)	—	—	—	178.99

**Figure 2:**
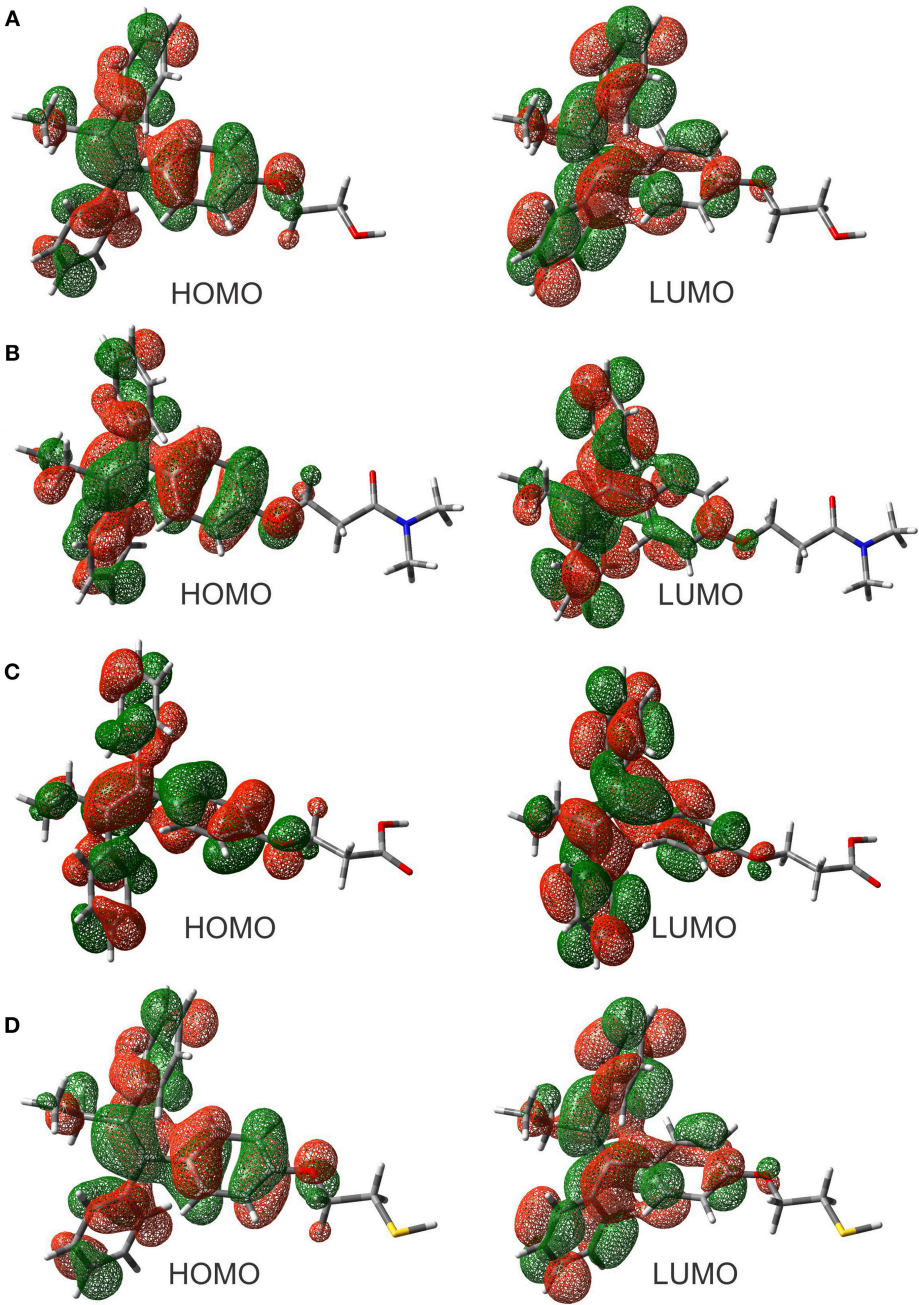
Highest occupied molecular orbitals (HOMO) and lowest unoccupied molecular orbitals (LUMO) of the **(A)** TAM-Hydroxyl, **(B)** TAM-Amide, **(C)** TAM-Carboxyl, and **(D)** TAM-Sulfhydryl calculated at the M06/6-31G(d) level of theory.

**Figure 3:**
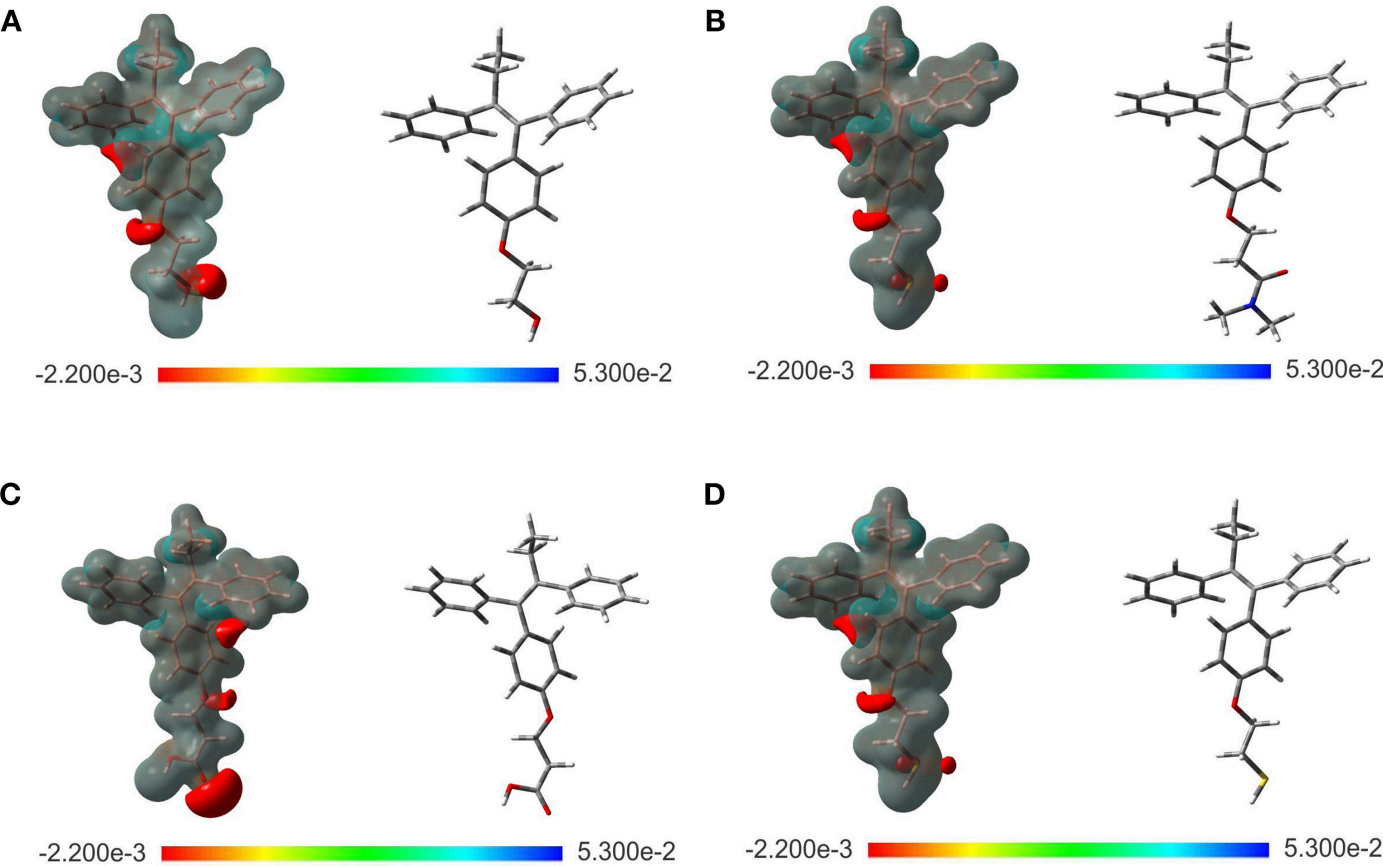
Optimized structure and electrostatic potential map on the molecular surface for **(A)** TAM-Hydroxyl, **(B)** TAM-Amide, **(C)** TAM-Carboxyl, and **(D)** TAM-Sulfhydryl calculated at the M06/6-31G(d) level of theory. Color range oscillates between −2.200e-3 to 5.300e-2: blue, more positive; red, more negative.

The evaluation of the highest occupied molecular orbital (HOMO) and the lowest unoccupied molecular orbital (LUMO) in each of the ligands were carried out to identify the zone that is richer in electrons. This analysis of the molecular orbitals allowed to explore the pharmacophore of the analogs. Figure 2 shows the HOMO and LUMO for the different ligands. In all cases, the pharmacophore of the TAM-analogs remains in the same area (phenyl, ethyl, and alkene functional groups) reported for the TAM drug (Landeros-Martínez et al., 2016). This study was also used to explain which zone of the ligands has the recognition ability in the hormone receptors. Furthermore, the electrostatic potential surface (EPS) maps were adequate for analyzing the binding sites on the basis of the recognition of one molecule by another, which is very important for drug design (Li et al., 2013). The maps in Figure 3 show in red the region with the most electronegative electrostatic potential and blue region for the most positive electrostatic potential. It can be observed that atoms that are more electronegative in TAM-Hydroxyl, TAM-Amide, and TAM-Carboxyl are the oxygen atoms while in TAM-Sulfihydryl are the oxygen and sulfur atoms.

#### 3.1.2. Reactivity parameters

Chemical reactivity parameters such as electron affinity, ionization potential, chemical hardness, electronegativity, chemical potential, and electrophilicity index obtained with energy differences approximation as well as HOMO-LUMO approximation are presented in Table 2. These values suggest that TAM-Amide has the greater ease to react in the presence of the hormonal receptors according to the chemical hardness in both approximations. Also, the electrophilicity index information allowed to determinate that all TAM-analogs are strong electrophiles with ω > 1.5 eV for both approximations in accordance with Domingo et al. (2016). In addition, the nucleophilicity index was calculated by

(5) $$\begin{array}{l} N_{\left(Nu\right)}=E_{HOMO\left(Nu\right)}\left(eV\right)-E_{HOMO\left(TCE\right)}\left(eV\right) \end{array}$$

Tetracyanoethylene (TCE) was used as a reference for these scales of nucleophilicity because it presents the lowest HOMO energy in a large series of molecules previously studied, being the E_*HOMO*_ of the TCE of −9.13 eV. The values of nucleophilicity of the TAM drug, TAM-Hydroxyl, TAM-Amide, TAM-Carboxyl, and TAM-Sulfhydryl are 3.42, 3.40, 3.43, 3.41, and 3.38 eV, respectively. All the molecules have a strong nucleophiliic character with N > 3.0 eV according to the scale proposed by Domingo et al. (2016).

**Table 2:** Reactivity parameters of the TAM-analogs determined at the M06/6-31G(d) level of theory with energy differences and HOMO-LUMO approximations.

**TAM-analogs**	**A (eV)**	**I (eV)**	**η (eV)**	**χ = −μ (eV)**	**ω (eV)**
TAM-Hydroxyl	1.08 / 0.75	5.48 / 5.73	2.20 / 2.49	3.28 / 3.24	2.45 / 2.11
TAM-Amide	1.10 / 0.77	5.45 / 5.70	2.17 / 2.46	3.27 / 3.23	2.46 / 2.12
TAM-Carboxyl	1.10 / 0.77	5.45 / 5.72	2.19 / 2.48	3.29 / 3.24	2.47 / 2.13
TAM-Sulfhydryl	1.09 / 0.77	5.49 / 5.74	2.20 / 2.50	3.29 / 3.26	2.46 / 2.13

Another important result of the TAM-analogs is the molecular polar surface area (PSA), which allows the prediction of the transport properties of drugs through membranes. PSA consists of the sum of all polar atoms, including the oxygen, nitrogen and hydrogen attached to these atoms (Ertl, 2008). The results were 29.46 , 29.54 46.53 , and 9.63 Å^2^ for TAM-Hydroxyl, TAM-Amide, TAM-Carboxyl, and TAM-Sulfhydryl, respectively. According to Clark, the drugs with a value less than 90 Å^2^ are completely absorbed in the cell membranes, while those drugs with values greater than 140 Å^2^ are poorly cell permeable (Clark, 1999).

### 3.2. Analysis of the hormone receptors with the tamoxifen analogs

**Figure 4:**
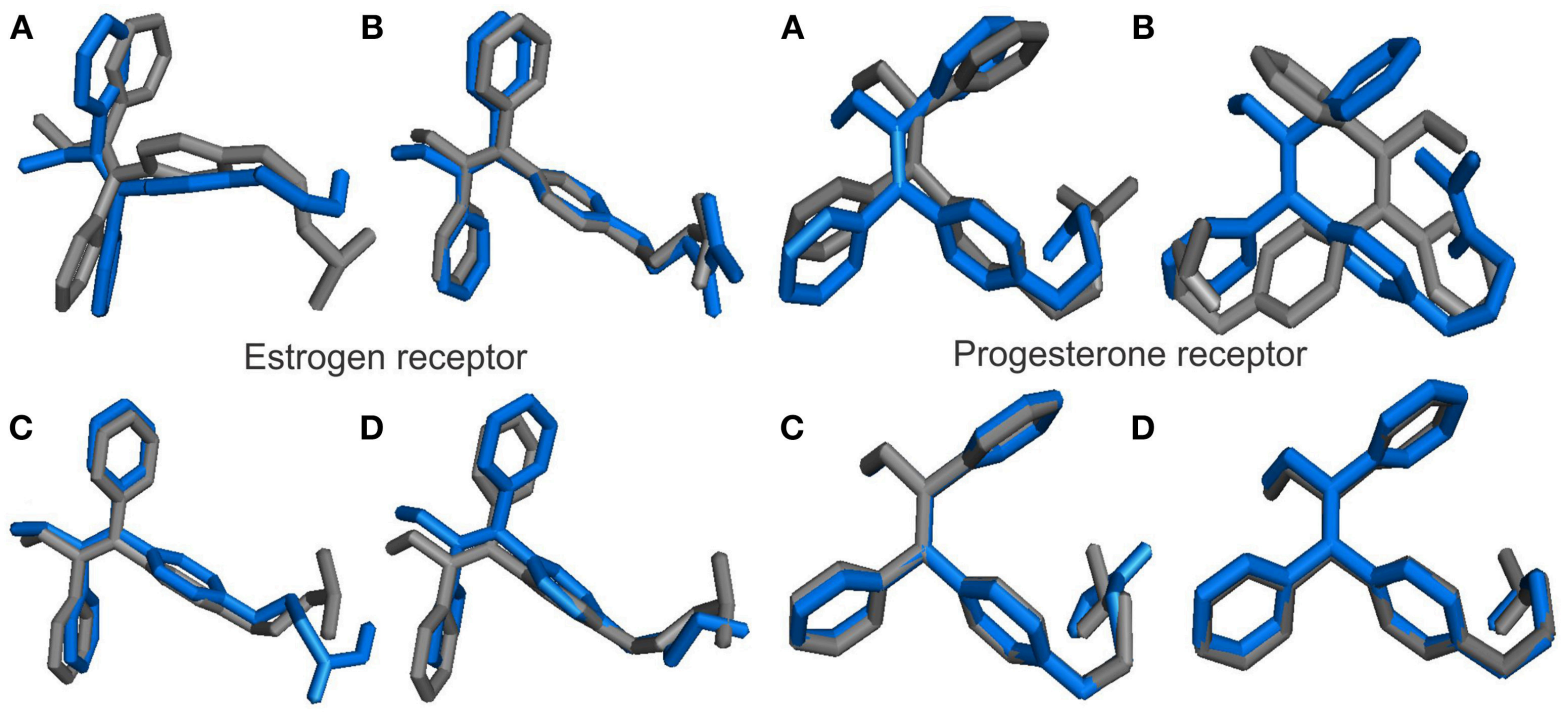
Alignments between the co-crystal structure TAM (gray) and the TAM-analogs (blue) at their absolute positions in the binding pocket in hormone receptors for **(A)** TAM-Hydroxyl, **(B)** TAM-Amide, **(C)** TAM-Carboxyl, and **(D)** TAM-Sulfhydryl.

The binding modes of a series of TAM-analogs were estimated by means of molecular docking calculations. The value of the root mean square deviation (RMSD) was considered as a measure of the accuracy of the docking results. The optimal RMSD value must be lower than 2 Å (Samanta and Das, 2016). Figure 4 shows the alignments to the native co-crystallized structure TAM (gray) with each one TAM-analogs (blue). Therefore, the RMSD in the estrogen receptor obtained between TAM with TAM-Hydroxyl, TAM-Amide, TAM-Carboxyl, and TAM-Sulfhydryl are 3.03 , 1.824 , 19.67 , and 2.272 Å, respectively, while for the progesterone receptor the RMSD value between TAM and TAM-Hydroxyl, TAM-Amide, TAM-Carboxyl, and TAM-Sulfhydryl are 2.082 , 3.445, 0.148, and 0.949 Å.

#### 3.2.1. Docking analysis of the estrogen receptor

All the TAM-analogs were successfully docked into the binding pocket of ER. In this work, the attention has been focused on the estrogen receptor-ligand because this analysis allows to determine which of these analogs are the most or least active.

**Figure 5:**
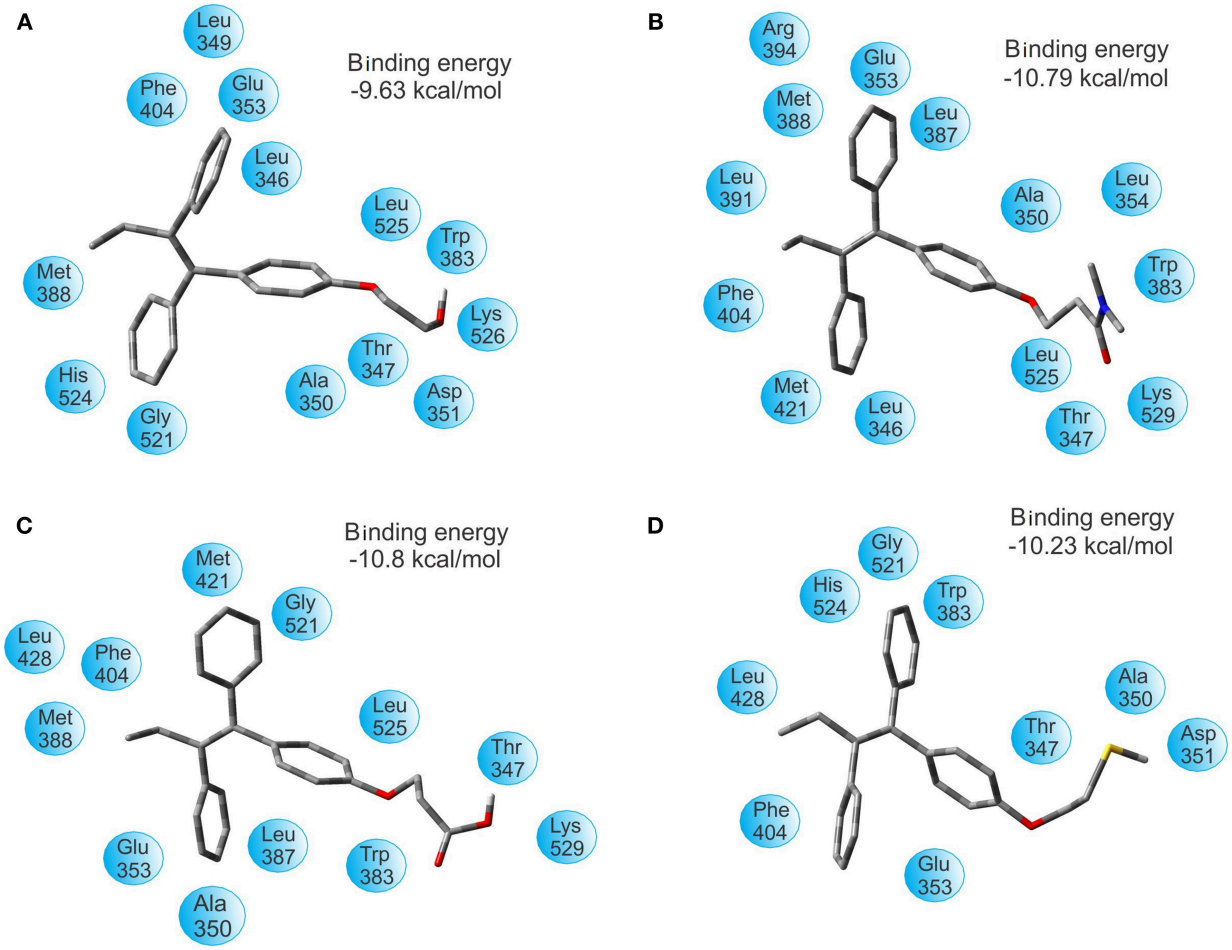
Optimal docking position and binding energy of the estrogen receptor with **(A)** TAM-Hydroxyl, **(B)** TAM-Amide, **(C)** TAM-Carboxyl, and **(D)** TAM-Sulfhydryl.

The binding energy of TAM-Hydroxyl, TAM-Amide, TAM-Carboxyl, and TAM-Sulfhydryl with the ER are −9.63 , −10.79, −10.80, and −10.23 kcal/mol, respectively. Figure 5 shows the optimal docking position and binding energy into the binding pocket of ER. It has been observed that each of the TAM-analogs is located at the pocket site of the ER. Furthermore, based on our previous experience, it can be said that in spite of the differences between the ΔG values in each case being small, the results of the binding energies are significant enough to assert that the TAM-Amide and TAM-Carboxyl species are the most active while the least active is TAM-Hydroxyl in the pocket site of the ER.

**Figure 6:**
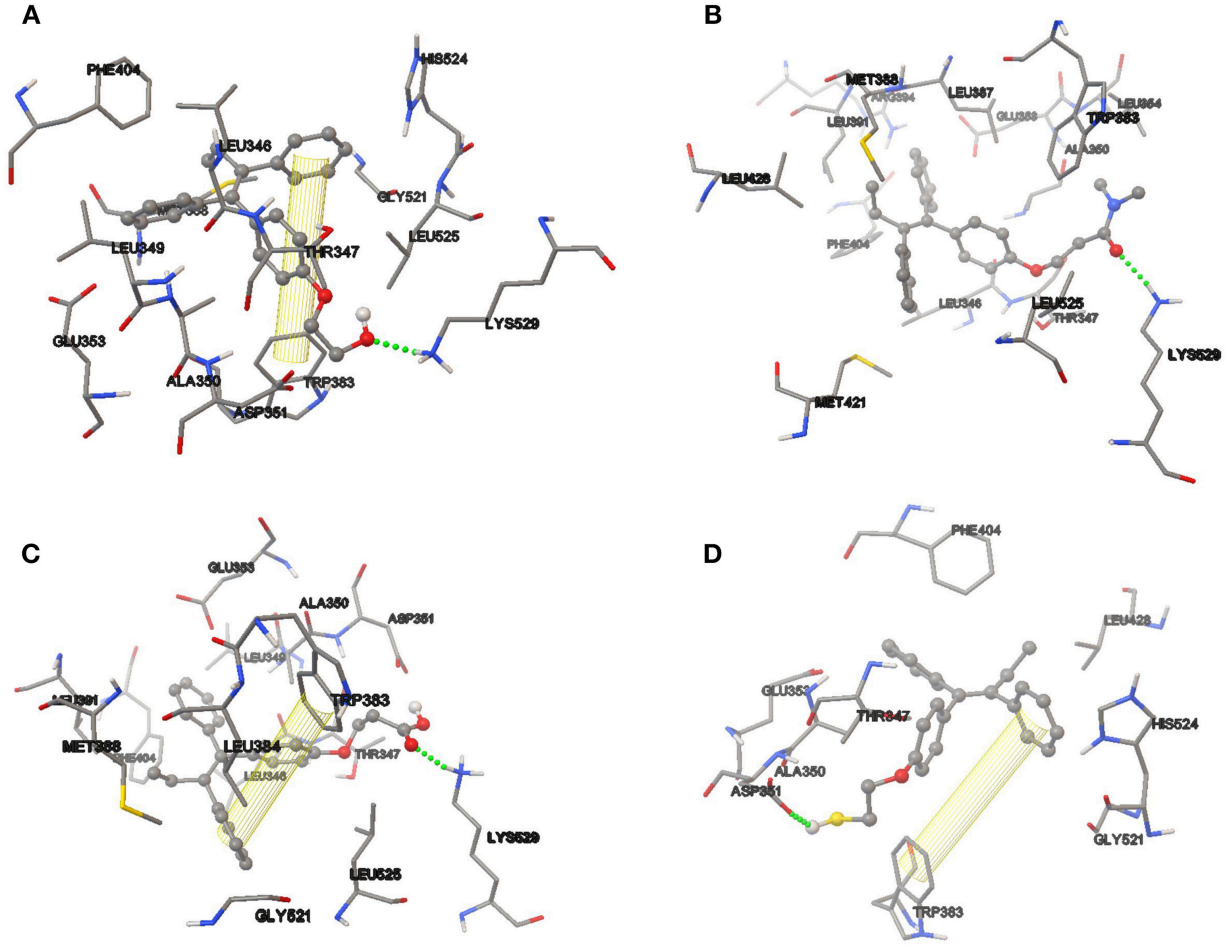
Hydrogen bond and π-π interactions at the active site of the estrogen receptor with **(A)** TAM-Hydroxyl, **(B)** TAM-Amide, **(C)** TAM-Carboxyl, and **(D)** TAM-Sulfhydryl.

After successful analysis of the bonding mode of TAM analogs, the hydrogen bond and π-π interaction were analyzed in each of the couplings. TAM-Hydroxyl has one π-π interaction and one hydrogen bond between hydroxyl of the analog and the oxygen atom of Lys 529 (OH–O, 1.94 Å); TAM-Amide formed one hydrogen bond with the oxygen atom of the ligand and the NH of Lys 529 (O–NH, 2.078 Å); TAM-Carboxyl present one π-π interaction and one hydrogen bond between oxygen atom of the analog and NH of Lys 529 (O–NH, 1.845 Å) and finally TAM-Sulfhydryl has one π-π interaction and one hydrogen bond between sulfhydryl (SH) of the ligand and the oxygen atom of Asp 351 (SH–O, 1.759 Å). In all cases, the TAM-analogs analyzed follow the Lipinsky Rule of Five which is used to predict whether a compound has or not has a drug-like character (Leeson, 2012). Additionally, when there are five or fewer hydrogen bonds, it can be said that the drug have good absorption or permeation and will be more active (Lipinski et al., 2001). Figure 6 shows the TAM-analogs in ball and stick and the amino acids of the pocket site in tube. The hydrogen bonds are showed in green dots and π-π interactions are the area in yellow.

#### 3.2.2. Reactivity parameters

The values of reactivity parameters calculated for each of the TAM-analogs and the amino acids of the pocket site of the ER were estimated using the vertical A and I and are given in Tables 3, 4 respectively. The chemical potential of the TAM-analogs varies from −3.23 to −3.44 eV. Meanwhile, for the active site in each of the couplings, the values range from −2.39 to −4.31 eV. For the amino acids of the active site, the electronegativity decreases in the order Leu 346-Thr 347 > Lys 529 > Leu 525 > Thr 347 > Leu 387-Met 388 > Arg 394 > Leu 428 > Ala 350 > Leu 428 > Gly 521 > Met 421 > Phe 404 > Leu 387-Met 388 > His 524 > Leu 349-Ala 350-Asp 351 >Trp 383 > Glu 353- Leu354 > Ala 350-Asp 351 > Glu 353 > Met 388. The maximum value of electronegativity of the TAM-analogs is for TAM-Carboxyl; therefore the maximum difference in electronegativity occurs between TAM-Carboxyl and Glu 353.

**Table 3:** Reactivity parameters of the different TAM analogs in the pocket size of the estrogen receptor.

**TAM-analogs**	**A (eV)**	**I (eV)**	**η (eV)**	**χ = −μ (eV)**	**ω (eV)**
TAM-Hydroxyl	0.66	5.81	2.57	3.23	2.03
TAM-Amide	0.86	5.90	2.54	3.37	2.46
TAM-Carboxyl	0.74	6.15	2.71	3.44	2.19
TAM-Sulfhydryl	0.74	6.03	2.64	3.38	2.17

**Table 4:** Reactivity parameters of the pocket site of the estrogen receptor.

**TAM-analogs**	**Active site**	**A (eV)**	**I (eV)**	**η (eV)**	**χ = −μ (eV)**	**ω (eV)**
	Phe 404	0.51	6.40	2.95	3.46	2.03
	Leu 346-Thr 347	0.88	7.74	3.43	4.31	2.71
	Glu 353	0.20	5.62	2.71	2.91	1.57
	Leu 349-Ala 350 -Asp 351	0.43	2.79	3.53	3.26	2.10
TAM-Hydroxyl	Lys 529	0.83	7.22	3.19	4.02	2.54
	Trp 383	0.67	5.81	2.57	3.24	2.04
	His 524-Leu 525	0.81	6.10	2.65	3.46	2.25
	Gly 521	0.21	7.03	3.41	3.62	1.92
	Met 388	0.46	6.11	2.82	2.39	1.91
	Arg 394	0.41	7.10	3.34	3.75	2.11
	Leu 391	0.27	6.21	2.97	3.24	1.77
	Phe 404	0.51	6.40	2.95	3.46	2.03
	Leu 428	0.47	7.00	3.23	3.73	2.14
	Met 421	0.88	6.18	2.65	3.53	2.35
TAM-Amide	Leu 387-Met 388	0.51	6.25	2.87	3.38	1.99
	Leu 346-Thr 347	0.88	7.74	3.43	4.31	2.71
	Glu 353-Leu 354	0.66	5.59	2.47	3.13	1.98
	Ala 350	0.31	7.09	3.39	3.70	2.02
	Trp 383	0.67	5.81	2.57	3.24	2.04
	Leu 525	0.82	7.03	3.10	3.93	2.48
	Lys 529	0.83	7.22	3.19	4.02	2.54
	Leu 428	0.47	7.00	3.23	3.73	2.14
	Phe 404	0.51	6.40	2.95	3.46	2.03
	Leu 387-Met 388	0.51	6.25	2.87	3.87	1.99
	Glu 353	0.20	5.62	2.71	2.91	1.57
	Ala 353	0.31	7.09	3.39	3.70	2.02
TAM-Carboxyl	Trp 383	0.67	5.81	2.57	3.24	2.04
	Thr 347	0.62	7.21	3.30	3.92	2.32
	Lys 529	0.83	7.22	3.19	4.02	2.54
	Leu 525	0.82	7.03	3.10	3.93	2.48
	Gly 521	0.21	7.03	3.41	3.62	1.92
	Met 421	0.88	6.18	2.65	3.53	2.35
	Phe 404	0.51	6.40	2.95	3.46	2.03
	Glu 353	0.20	5.62	2.71	2.91	1.57
	Leu 428	0.47	7.00	3.33	3.67	2.02
TAM-Sulfhydryl	Gly 521	0.21	7.03	3.41	3.62	1.92
	His 524	0.43	6.20	2.89	3.31	1.90
	Trp 383	0.67	5.81	2.57	3.24	2.04
	Thr 347	0.62	7.21	3.30	3.92	2.32
	Ala 350-Asp 351	0.45	5.77	2.66	3.11	1.81

Among TAM-analogs, the TAM-Amide has the lowest chemical hardness which means this molecule is more reactive in the presence of ER. The chemical hardness of the TAM-analogs are in the order: TAM-Amide > TAM-Hydroxyl > TAM-Sulfhydryl > TAM-Carboxyl. The chemical hardness of the active site of the four couplings are in the order: Glu 353- Leu 354 > Trp 383 > Met 421 > Ala 350-Asp 351 > Glu 353 >Met 388 > Leu 387-Met 388 > His 524 > Phe 404 >Leu391 > Leu 525 > Lys 529 > Leu 428 > Thr 347 > Leu 428 > Arg 394 > Ala 350 > Gly521 > Leu 346-Thr 347 > Leu 349-Ala 350- Asp 351. The electrophilicity index suggest that TAM-Amide has a greater capacity to accept electrons from the pocket site, whereas in the pocket site of the couplings decreases in the order Leu 346-Thr 347 > Lys 529 > Leu 525 > Met 421 > Thr 347 > His 524-Leu 525 >Leu 428 > Arg 394 > Leu 349-Ala 350- Asp 351 > Trp 383 > Phe 404 > Leu 428 > Leu 387-Met 388 > Glu 353-Leu354 > Gly 521 > Met 388 >His 524 > Ala 350-Asp 351> Leu391 > Glu 353.

#### 3.2.3. Charge transfer in the estrogen receptor

The interaction between the TAM-analogs and the amino acids of the pocket site was calculated by means of the parameter ΔN which determines the fractional number of electrons transferred form a system A to a system B with ΔN described by Equation (4). In this formula, μ_*A*_ is for the TAM-analogs and μ_*B*_ is for the amino acids of the active site. η_*A*_ and η_*B*_ represent the chemical hardness of the TAM-analogs and the amino acids of the active site, respectively. Values of ΔN < 0 suggest that the charge transfer flows from A to B (A acts as an electron donor), and for values of ΔN > 0 charge flows from B to A (A acts as electron acceptor). In previous works, Kanvah et al. and Wan et al. have used the charge transfer concept to describe the oxidative damage in DNA bases (Wan et al., 2000; Kanvah and Schuster, 2005).

According to the results of Table 5, some amino acids of the pocket site act as electron donor for example: TAM-Hydroxyl with Glu 353 and Met 388, TAM-Amide with Leu 391, Phe 404, Glu 353-Leu354 and Trp 383, TAM-Carboxyl with Glu 353, and Trp 383 and finally TAM-Sulfhydryl with Glu 353, His 524, Trp 383, and Ala 350-Asp 351, while the rest of the amino acids act as electron acceptors. The oxidative damage in the active site decreases in the order TAM-Amide > TAM-Sulfhydryl > TAM-Hydroxyl >TAM-Carboxyl.

**Table 5:** Charge transfer between TAM analogs and the estrogen receptor.

**TAM-analogs**	**Pocket site**	**ΔN**
	Phe 404	−0.021
	Leu 346-Thr 347	−0.090
	Glu 353	0.030
	Leu 349-Ala 350-Asp 351	−0.002
TAM-Hydroxyl	Lys 529	−0.069
	Trp 383	−0.001
	His 524-Leu 525	−0.022
	Gly 521	−0.033
	Met 388	0.078
	Arg 394	−0.032
	Leu 391	0.012
	Phe 404	0.062
	Leu 428	−0.031
	Met 421	−0.015
TAM-Amide	Leu 387-Met 388	−0.001
	Leu 346-Th 347	−0.079
	Glu 353-Leu 354	0.024
	Ala 350	−0.028
	Trp 383	0.013
	Leu 525	−0.050
	Lys 529	−0.057
	Leu 428	−0.024
	Phe 404	−0.002
	Leu 387-Met 388	−0.039
	Glu 353	0.049
	Ala 350	−0.021
TAM-Carboxyl	Trp 383	0.019
	Thr 347	−0.040
	Lys 529	−0.049
	Leu 525	−0.042
	Gly 521	−0.015
	Met 421	−0.008
	Phe 404	−0.007
	Glu 353	0.044
	Leu 428	−0.024
TAM-Sulfhydryl	Gly 521	−0.020
	His 524	0.006
	Trp 383	0.013
	Thr 347	−0.045
	Ala 350-Asp 351	0.025

#### 3.2.4. Docking analysis of the progesterone receptor

**Figure 7:**
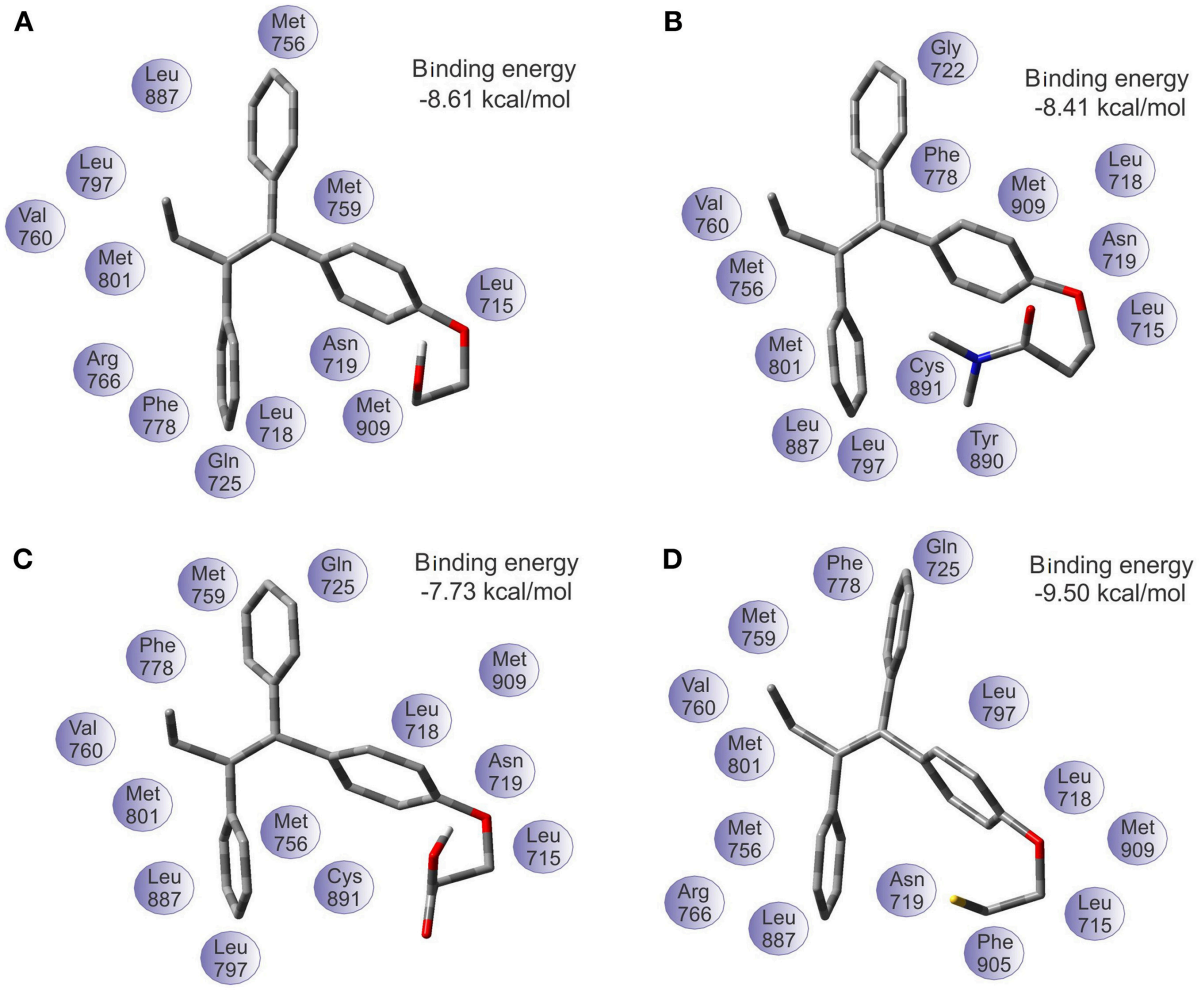
Optimal docking position and binding energy of the progesterone receptor with **(A)** TAM-Hydroxyl, **(B)** TAM-Amide, **(C)** TAM-Carboxyl, and **(D)** TAM-Sulfhydryl.

The binding energy values of progesterone receptor (PR) are: −8.61 kcal/mol for TAM-Hydroxyl, −8.41 kcal/mol for TAM-Amide, −7.73 kcal/mol for TAM-Carboxyl, and −9.50 kcal/mol for TAM-Sulfhydryl. Figure 7 shows the most favorable docking positions and binding energies into the binding pocket of PR. Here the situation is simpler to understand in comparison with the case of the estrogen receptor. According to the results of the binding energies, the TAM-Sulfhydryl is the most active while TAM-Carboxyl is the least active in the pocket site of the PR.

**Figure 8:**
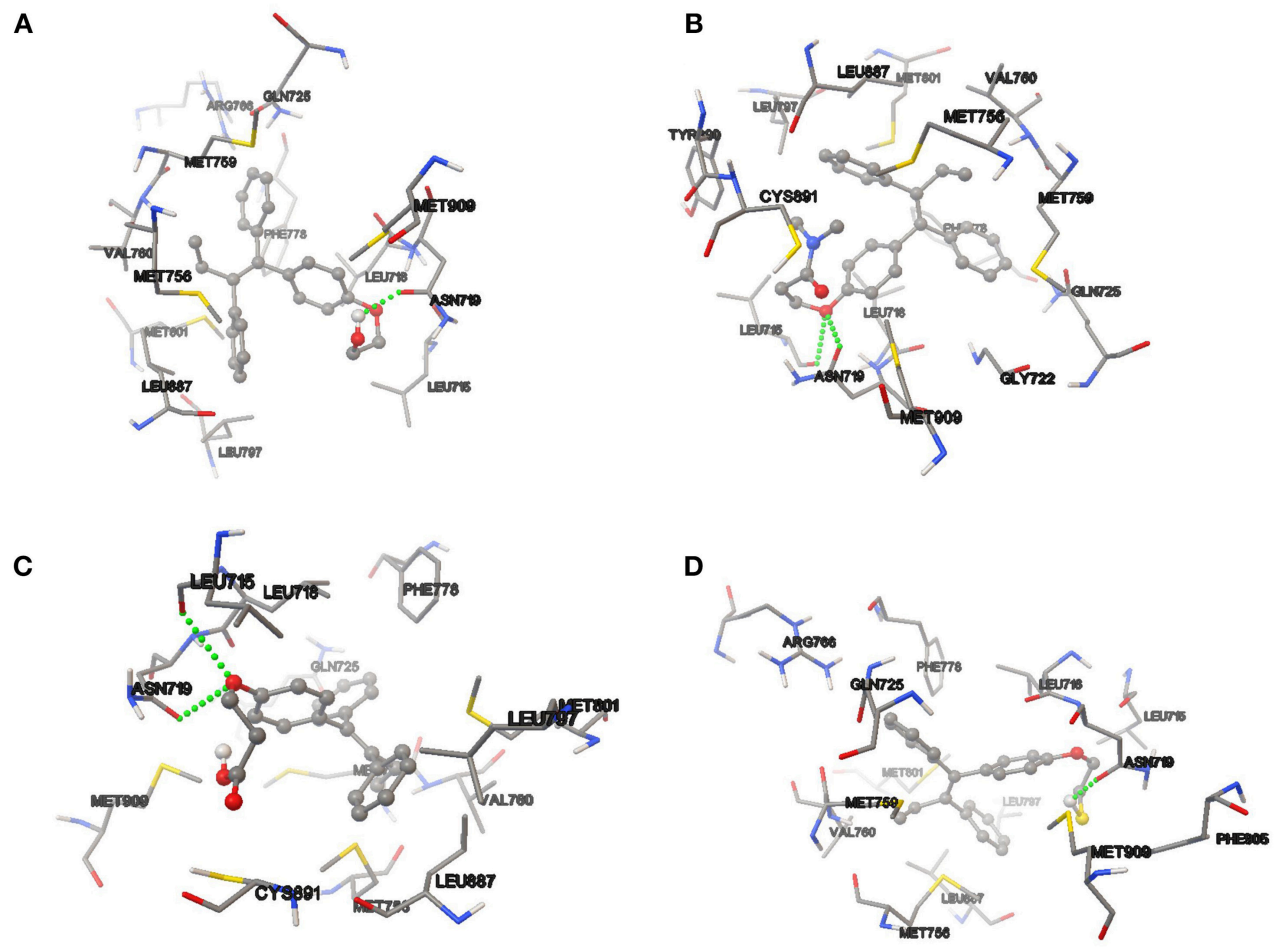
Hydrogen bond and π-π interactions at the active site of the progesterone receptor with **(A)** TAM-Hydroxyl, **(B)** TAM-Amide, **(C)** TAM-Carboxyl, and **(D)** TAM-Sulfhydryl.

On the other hand, the analysis of hydrogen bond and π-π interaction in each of the couplings showed that TAM-Hydroxyl formed one hydrogen bond between hydroxyl of the analog and the oxygen atom of Asn 719 (OH–O, 1.971 Å); TAM-Amide has two hydrogen bonds with either of the ligand and the oxygen atom of Leu 715 (O–O, 3.046 Å) and Asn 719 (O–O, 2.449 Å); TAM-Carboxyl present two hydrogen bonds between either of the analog and oxygen of Leu 715 (O–O, 2.984 Å) and Asp 719 (O–O, 2.705 Å). Meanwhile TAM-Sulfhydryl has one hydrogen bond between sulfhydryl (SH) of the ligand and the oxygen atom of Asn 719 (SH–O, 1.886 Å). All TAM-analogs have good absorption or permeation according to the Lipinsky Rule of Five (Lipinski et al., 2001). Figure 8 shows the TAM-analogs in ball and stick and the amino acids of the pocket site in tube. The hydrogen bonds are showed in green dots.

#### 3.2.5. Reactivity parameters

After having obtained the most stable structure of TAM-analogs in the pocket site, an analysis of the chemical reactivity of TAM-analogs and progesterone residues was performed by means of the reactivity descriptors. The results for these calculations are presented in Tables 6, 7, respectively.

**Table 6:** Reactivity parameters of the different TAM-analogs in the pocket size of the progesterone receptor.

**TAM-analogs**	**A (eV)**	**I (eV)**	**η (eV)**	**χ = −μ (eV)**	**ω (eV)**
TAM-Hydroxyl	0.93	5.99	2.53	3.46	2.36
TAM-Amide	1.01	5.63	2.31	3.32	2.39
TAM-Carboxyl	1.00	6.20	2.60	3.40	2.49
TAM-Sulfhydryl	1.00	5.92	2.46	3.46	2.43

**Table 7:** Reactivity parameters of the pocket site of the progesterone receptor.

**TAM-analogs**	**Active site**	**A (eV)**	**I (eV)**	**η (eV)**	**χ = −μ (eV)**	**ω (eV)**
	Met 756	0.75	6.30	2.77	3.52	2.54
	Leu 887	0.43	7.01	3.29	3.72	2.10
	Leu 797	0.67	6.86	3.09	3.76	2.29
	Met 801	0.64	6.27	2.82	3.45	2.12
TAM-Hydroxyl	Arg 766	0.78	6.70	2.96	3.74	2.36
	Phe 778	0.86	6.60	2.87	3.73	2.42
	Gln 725	0.70	7.10	3.20	3.90	2.38
	Leu 715	0.60	7.02	3.21	3.81	2.26
	Met 909	0.50	6.21	2.85	3.36	1.97
	Met 759-Val 760	1.05	6.26	2.61	3.65	2.56
	Leu 718-Asn 719	1.06	7.06	3.00	4.06	2.74
	Leu 887	0.43	7.01	3.29	3.72	2.10
	Met 801	0.64	6.27	2.82	3.45	2.12
	Met 756	0.75	6.30	2.77	3.52	2.54
	Phe 778	0.86	6.60	2.87	3.73	2.42
	Gln 725	0.70	7.10	3.20	3.90	2.38
TAM-Amide	Gly 772	0.51	6.25	2.87	3.38	1.99
	Met 909	0.50	6.21	2.85	3.36	1.97
	Leu 715	0.60	7.02	3.21	3.81	2.26
	Leu 797	0.67	6.86	3.09	3.76	2.29
	Met 759-Val 760	1.05	6.26	2.61	3.65	2.56
	Leu 718-Asn 719	1.06	7.06	3.00	4.06	2.74
	Tyr 890-Cys 891	0.74	6.06	2.66	3.40	2.17
	Met 801	0.64	6.27	2.82	3.45	2.12
	Phe 778	0.86	6.60	2.87	3.73	2.42
	Gln 725	0.70	7.10	3.20	3.90	2.38
	Met 909	0.50	6.21	2.85	3.36	1.97
	Cys 891	0.55	6.89	3.17	3.72	2.18
TAM-Carboxyl	Met 756	0.75	6.30	2.77	3.53	2.24
	Leu 797	0.67	6.86	3.09	3.76	2.29
	Leu 887	0.43	7.01	3.29	3.72	2.10
	Met 759-Val 760	1.05	6.26	2.61	3.65	2.56
	Leu 718-Asn 719	1.06	7.06	3.00	4.06	2.74
	Phe 778	0.86	6.60	2.87	3.73	2.42
	Met 756	0.75	6.30	2.77	3.53	2.24
	Arg 766	0.78	6.70	2.96	3.74	2.36
	Leu 887	0.43	7.01	3.29	3.72	2.10
	Leu 797	0.67	6.86	3.09	3.76	2.29
TAM-Sulfhydryl	Phe 905	0.93	6.58	2.82	3.76	2.50
	Leu 715	0.60	7.02	3.21	3.81	2.26
	Met 909	0.50	6.21	2.85	3.36	1.97
	Gln 725	0.70	7.10	3.20	3.90	2.38
	Met 801	0.64	6.27	2.82	3.45	2.12
	Met 759-Val 760	1.05	6.26	2.61	3.65	2.56
	Leu 718-Asn 719	1.06	7.06	3.00	4.06	2.74

The highest value of the electron affinity in the TAM-analogs is TAM-Amide and in the pocket site of each of the couplings is for the Leu 718-Asn 719 residue. The ionization potential results show that the greatest possibility of losing electrons in the TAM-analogs is TAM-Carboxyl and in the amino acids of the pocket site in each of the couplings is for the Leu 718-Asn 719 residue. The chemical hardness of the TAM-analogs are in the order: TAM-Amide > TAM-Sulfhydryl > TAM-Hydroxyl > TAM-Carboxyl. The chemical hardness of the pocket site in each of the couplings is in the order: Met 759-Val 760 > Tyr89- Cys 891 > Met 756 > Met 801 > Phe 905 > Met 909 > Phe 778 > Gly 722 > Arg 766 > Leu 718-Asn 719 > Leu 797 > Cys 891 > Gln 725 > Leu 715 > Leu 887. Among the TAM-analogs, the electronegativity decreases in the order TAM-Hydroxyl > TAM-Sulfhydryl > TAM-Carboxyl > TAM-Amide. As the minimum value of the electronegativity within the pocket site in the coupling of TAM-Hydroxyl is for Met 909. Therefore, the maximum difference in electronegativity occurs in this case between TAM-Hydroxyl and Met 909. The values for the electrophilicity index of the TAM-analogs indicate that TAM-Carboxyl have the greatest capacity to accept electrons of the pocket site, while the residues of each of the couplings decrease in the order: Leu 718-Asn 719 > Met 759-Val 760 > Met 756 > Phe 905 > Phe 778 > Gln 725 > Arg 766 > Leu 797 > Leu 715 > Met 756 > Cys 891 > Tyr89-Cys 891> Met 801 > Leu 887 >Gly 722 > Met 909.

#### 3.2.6. Charge transfer in the progesterone receptor

The amount of charge transfer between the TAM-analogs and the amino acids of the pocket site was estimated with the parameter ΔN described with Equation (4). The values of ΔN are shown in Table 8. The analysis of the interaction of TAM-Hydroxyl with Met 801 and Met 909, TAM-Amide with Met 909, TAM-Carboxyl with Met 909, and TAM-Sulfhydryl with Met 801 and Met 909 for the charge transfer is positive indicating that these TAM analogs act as electron acceptors. Meanwhile, the one with the rest of amino acids acts as electron donor. The oxidative damage in the pocket site decreases in the order: TAM-Sulfhydryl > TAM-Hydroxyl > TAM-Amide > TAM-Carboxyl.

**Table 8:** Charge transfer between TAM analogs and the progesterone receptor.

**TAM-analogs**	**Pocket site**	**ΔN**
	Met 756	−0.006
	Leu 887	−0.022
	Leu 797	−0.027
	Met 801	0.001
	Arg 766	−0.026
TAM-Hydroxyl	Phe 778	−0.025
	Gln 725	−0.038
	Leu 715	−0.030
	Met 909	0.009
	Met 759-Val 760	−0.034
	Leu 718-Asn 719	−0.070
	Leu 887	−0.036
	Met 801	−0.013
	Met 756	−0.020
	Phe 778	−0.040
	Gln 725	−0.053
TAM-Amide	Gly 722	−0.006
	Met 909	−0.004
	Leu 715	−0.044
	Leu 797	−0.041
	Met 759-Val 760	−0.034
	Leu 718-Asn 719	−0.059
	Tyr 890-Cys 891	0.000
	Met 801	−0.005
	Phe 778	−0.030
	Gln 725	−0.043
	Met 909	0.004
	Cys 891	−0.028
TAM-Carboxyl	Met 756	−0.012
	Leu 797	−0.032
	Leu 887	−0.027
	Met 759-Val 760	−0.024
	Leu 718-Asn 719	−0.055
	Phe 778	−0.025
	Met 756	−0.007
	Arg 766	−0.026
	Leu 887	−0.022
	Leu 797	−0.027
TAM-Sulfhydryl	Phe 905	−0.028
	Leu 715	−0.031
	Met 909	0.009
	Gln 725	−0.039
	Met 801	0.001
	Met 759-Val 760	−0.019
	Leu 718-Asn719	−0.055

## 4. Conclusions

In this work, the replacement of polar groups, such as the hydroxyl, amide, carboxyl, and sulfhydryl in the hydrophilic zone of the TAM drug did not modified the pharmacophore. According to the PSA values, the permeability in cell of the TAM-analogs decreases in the order: TAM-Sulfhydryl > TAM-Hydroxyl > TAM-Amide > TAM-Carboxyl. The scale of electrophilicity of Domingo et al. allowed to classify all TAM-analogs as strong electrophiles.

The coupling of ER with each of the TAM-analogs showed that TAM-Carboxyl and TAM-Amide are the most active in the pocket site while TAM-Hydroxyl is the least active in the pocket site and in both cases the couplings have one hydrogen bond and one π-π interaction. According to the charge transfer descriptor, the coupling ER-TAM-Sulfhydryl and ER-TAM-Amide presented the greatest oxidative damage. In turn, the coupling of PR with the TAM-analogs showed that the most active analog is TAM-Sulfhydryl and the least active is TAM-Carboxyl, presenting in both cases one hydrogen bond. The charge transfer descriptor shows that the TAM-Sulfhydryl and TAM-Hydroxyl are more damage oxidative in the pocket site of the PR. The four TAM-analogs have a good biodistribution, permeability, and pharmacological action on the hormone receptors, according to the Lipinsky Rule of Five.

The values of the chemical hardness for TAM into the pocket site of ER and PR have been calculated earlier by us as being 2.40 and 2.33 eV, respectively. Thus, according with the chemical hardness values, TAM has a greater ease to react than the analogs in presence of both hormonal receptors. We can conclude that the activity has been not improved with any of the the TAM-analogs.

If we consider the selectivity or the degree to which the analogs acts in the active site, TAM-amide and TAM-carboxyl analogs improved the binding energy regarding with TAM in less than 0.5 kcal/mol for the case of the ER receptor for which was calculated as −10.38 Kcal/mol. In turn, for the PR case, there is an improvement in the binding energy exclusively with TAM-Sulfhydryl with −9.50 kcal/mol compared with −9.38 Kcal/mol of TAM calculated previously. However, due to the small difference between the two values, it can be concluded that this is a rather limited improvement. The main conclusion is that a marked better activity and selectivity improvement is not achieved through the studied TAM-analogs.

The reasoning behind the election of the different radical groups for building the different TAM-analogs was based on the previous knowledge of the improvement in the binding energy of hydroxyl-TAM metabolites. Nevertheless, the improvement was not significant. We believe that this behavior can be related with the low number of H-bonds because the studied TAM-analogs have only one of these bonds with either of the receptors. For this reason, the future design of potential TAM-analogs should include radical groups that make easier the formation of these kind of bonds.

Moreover, it is of outermost importance to increase the electron donor ability of the ligands and this could be probably achieved by including radical groups containing a larger number of polar atoms.

Finally, although the number of π-π bonds need to be larger in order to improve the interaction of the receptors with the TAM-analogs, this is not a fundamental issue because that interaction takes place between the rings of the pharmacophore and the receptor and our intention is to modify only the hydrophilic functional group.

## Author contributions

L-LL-M, NF-H, and DG-M conceived and designed the research and equally headed, wrote, and revised the manuscript.

### Conflict of interest statement

The authors declare that the research was conducted in the absence of any commercial or financial relationships that could be construed as a potential conflict of interest.
