# Supplementary material for: Interaction of Tamoxifen Analogs With the Pocket Site of Some Hormone Receptors. A Molecular Docking and Density Functional Theory Study
Source: Front Chem. 2018 Jul 13;6:293. doi: 10.3389/fchem.2018.00293 (PMC6053509; doi:10.3389/fchem.2018.00293)
Supplement: Supplementary file 1 [file Data_Sheet_1.pdf]

Supplementary Material: Interaction of Tamoxifen Analogues with the Pocket Site of some Hormone Receptors. A Molecular Docking and Density Functional Theory Study

Linda-Lucero Landeros-Martínez, Daniel Glossman-Mitnik and Norma Flores-Holguín
e-mail: linda.landeros@cimav.edu.mx - daniel.glossman@cimav.edu.mx - norma.flores@cimav.edu.mx

Table S1. Cartesian coordinates (in Å) of the optimized molecular structure of the TAM-Hydroxyl analogue. Hydrogen atoms are not shown.

Atom	x	y	z
O1	-4.201	-0.381	0.460
C2	1.443	-0.394	0.096
C3	2.165	0.751	0.138
C4	-0.040	-0.407	0.203
C5	3.670	0.796	0.211
C6	2.067	-1.738	-0.018
C7	1.517	2.088	0.094
C8	-0.862	-0.155	-0.904
C9	-0.662	-0.630	1.433
C10	4.220	1.573	-0.999
C11	-2.845	-0.401	0.447
C12	-2.241	-0.152	-0.790
C13	-2.047	-0.641	1.566
C14	1.759	-2.608	-1.070
C15	2.952	-2.184	0.971
C16	1.396	2.874	1.246
C17	1.064	2.615	-1.121
C18	-6.155	-0.833	-0.762
C19	-4.877	-0.031	-0.731
C20	2.334	-3.873	-1.146
C21	3.534	-3.443	0.895
C22	0.809	4.135	1.190
C23	0.486	3.879	-1.181
C24	3.228	-4.293	-0.164
C25	0.354	4.641	-0.023
O49	-6.307	-1.396	0.520

Table S2. Cartesian coordinates (in Å) of the optimized molecular structure of the TAM-Amide analogue. Hydrogen atoms are not shown.

Atom	x	y	z
O1	2.817	-0.980	-1.681
C2	-2.417	0.482	-0.111
C3	-3.309	-0.433	0.340
C4	-1.033	0.120	-0.512
C5	-4.650	-0.081	0.932
C6	-3.041	-1.892	0.262
C7	-2.729	1.933	-0.204
C8	-0.704	-0.179	-1.843
C9	-0.019	0.019	0.442
C10	-4.590	0.202	2.431
C11	1.590	-0.617	-1.232
C12	0.583	-0.541	-2.201
C13	1.283	-0.336	0.100
C14	-2.883	-2.538	-0.968
C15	-2.873	-2.644	1.433
C16	-3.128	2.672	0.914
C17	-2.676	2.584	-1.444
C18	4.744	0.013	-0.778
C19	3.839	-1.195	-0.722
C20	-2.597	-3.900	-1.029
C21	-2.578	-4.001	1.375
C22	-3.442	4.024	0.804
C23	-2.983	3.934	-1.556
C24	-2.442	-4.635	0.142
C25	-3.367	4.660	-0.431
C49	5.918	-0.146	0.167
O50	6.491	-1.230	0.270
N51	6.298	0.952	0.875
C52	7.364	0.843	1.850
C53	5.622	2.233	0.853

Table S3. Cartesian coordinates (in Å) of the optimized molecular structure of the TAM-Carboxyl analogue. Hydrogen atoms are not shown.

Atom	x	y	z
O1	3.623	-0.048	0.632
C2	-1.999	-0.330	0.086
C3	-2.766	0.780	-0.045
C4	-0.520	-0.275	0.216
C5	-4.240	0.751	-0.355
C6	-2.568	-1.704	0.074
C7	-2.205	2.145	0.138
C8	0.288	-0.176	-0.917
C9	0.114	-0.271	1.469
C10	-4.444	0.729	-1.881
C11	2.284	-0.117	0.426
C12	1.676	-0.107	-0.831
C13	1.491	-0.194	1.575
C14	-2.393	-2.544	1.182
C15	-3.329	-2.176	-1.001
C16	-1.735	2.869	-0.964
C17	-2.071	2.711	1.411
C18	5.175	-1.071	-0.797
C19	4.381	-1.234	0.480
C20	-2.934	-3.825	1.200
C21	-3.877	-3.456	-0.982
C22	-1.169	4.129	-0.802
C23	-1.513	3.974	1.574
C24	-3.678	-4.286	0.116
C25	-1.060	4.689	0.469
O49	7.284	-0.817	0.252
O50	6.735	0.677	-1.327
C51	6.455	-0.297	-0.667

Table S4. Cartesian coordinates (in Å) of the optimized molecular structure of the TAM-Sulfhydryl analogue. Hydrogen atoms are not shown.

Atom	x	y	z
O1	-3.684	-1.532	0.927
C2	1.442	0.679	0.012
C3	2.503	-0.101	-0.303
C4	0.084	0.116	0.241
C5	3.848	0.436	-0.722
C6	1.523	2.159	0.112
C7	2.430	-1.584	-0.234
C8	-0.830	0.009	-0.810
C9	-0.312	-0.368	1.497
C10	4.956	-0.513	-0.229
C11	-2.476	-0.986	0.634
C12	-2.103	-0.523	-0.628
C13	-1.568	-0.910	1.695
C14	2.013	2.907	-0.965
C15	1.071	2.847	1.245
C16	2.615	-2.265	0.973
C17	2.107	-2.329	-1.375
C18	-5.328	-0.770	-0.565
C19	-4.824	-0.698	0.858
C20	2.082	4.293	-0.901
C21	1.132	4.235	1.310
C22	2.516	-3.652	1.032
C23	1.998	-3.713	-1.316
C24	1.642	4.965	0.238
C25	2.205	-4.380	-0.113
S49	-5.813	0.918	-1.086
